# The Prognostic Significance of the Serum p53 Protein Concentration in Chinese Patients with Non-Hodgkin Lymphoma

**DOI:** 10.5505/tjh.2012.57338

**Published:** 2012-12-05

**Authors:** Min Zhou, Ling Cen, Tao Chen, Rong Xiao, Jianhe Yang, Nai-ke Giang, Yan Zhang

**Affiliations:** 1 Changzhou Second People’s Hospital Affiliated Nanjing Medical University, Department of Hematology, China

**Keywords:** non-Hodgkin lymphoma, p53 protein, Karyotype

## Abstract

**Objective:** To investigate the prognostic significance of cytogenetic abnormalities, staging, patient factors, and the serum p53 protein concentration in Chinese non-Hodgkin lymphoma (NHL) patients.

**Material and Methods:** The study included 43 patients with NHL that were identified between August 2003 and December 2008. Patient clinical characteristics patients were determined based on morphological, immunohistochemical, and cytogenetic analysis, and the serum p53 protein concentration was measured quantitatively.

**Results:** Following conventional chemotherapy, the complete/partial remission (CR/PR) rate was significantly higher and overall survival (OS) was significantly longer in the patients with early-stage (stage I-II) lymphoma, normal karyotype, and a low serum p53 protein concentration than in those with advanced-stage (stage III-IV) lymphoma, cytogenetic abnormalities, and a high serum p53 protein concentration (≥0.35 U/mL). Bone marrow infiltration was also a predictor of poor response and OS. There weren’t any significant differences in disease remission between the male and female patients, older and younger patients (aged <70 years vs. ≥70 years), or B-cell lymphoma and T-cell lymphoma patients.

**Conclusion:** Staging is an effective means of assessing the severity of NHL. Cytogenetic examination can provide useful information for diagnosis, staging, and prognostication. The serum p53 protein level may be a potential prognostic marker in patients with NHL.

**Conflict of interest:**None declared.

## Abstract (tr)

**Amaç:** Çinli non-Hodgkin lenfoma (NHL) hastalarında sitogenetik anormallikler, evrelendirme, hasta faktörleri ve serum p53 proteini konsantrasyonunun prognostik önemini araştırmak.

**Gereç ve Yöntemler:** Çalışmaya Ağustos 2003 ile Aralık 2008 arasında tanımlanan 43 NHL hastası dahil edildi. Hasta klinik özellikleri morfolojik, immünohistokimyasal ve sitogenetik analiz temelinde belirlendi ve serum p53 proteini konsantrasyonu kantitatif olarak ölçüldü.

**Bulgular:** Geleneksel kemoterapi sonrasında erken dönem (evre I-II) lenfoma, normal karyotip ve düşük serum p53 protein konsantrasyonu olan hastalarda tam/kısmi remisyon oranı ileri dönem (evre III-IV) lenfoma, sitogenetik bozukluklar ve yüksek serum p53 proteini konsantrasyonu (≥0,35 U/mL) olan hastalara göreönemli ölçüde daha yüksek ve genel sağkalım önemli ölçüde daha uzundu. Kemik iliği infiltrasyonu da kötü cevap ve genel sağkalımın bir prediktörüydü. Erkek ve kadın hastalar, yaşlı ve genç hastalar (<70 yaş ve ≥70 yaş) veya B-hücreli lenfoma ve T-hücreli lenfoma hastaları arasında hastalık remisyonu açısından önemli farklar yoktu.

**Sonuç:** Evrelendirme NHL şiddetini değerlendirmenin etkin bir yoludur. Sitogenetik inceleme tanı, evrelendirme ve prognoz belirleme için faydalı bilgiler sunabilir. Serum p53 proteini düzeyi NHL hastalarında olası bir prognostik işaret olabilir.

## INTRODUCTION

Non-Hodgkin lymphoma (NHL) is a heterogeneous disease that is generally categorized as indolent or aggressive, based on the morphology and proliferation of cancerous cells [1,2]. Differences in clinical features and treatment responses are evaluated based on the morphological, histological, immunophenotypic, cytogenetic, genetic, and molecular features that affect and indicate the aggressiveness of the disease. Nonetheless, the critical events associated with disease progression remain poorly understood, and their prognostic relevance has yet to be fully validated. In addition, p53 protein is known to be a tumor suppressor that maintains genomic stability—either by inducing cell cycle arrest or apoptosis. Although some studies have proposed that p53 protein expression is involved in lymphomagenesis, no study has analyzed the serum p53 protein level in Chinese NHL patients. As such, the present study aimed to evaluate the prognostic significance of the karyotype detected at the time of diagnosis, staging, patient factors, and the serum p53 protein concentration, according to the complete/partial remission (CR/PR) rate and overall survival (OS) in a cohort of Chinese NHL patients. In addition, the potential relationships between these factors were also evaluated.

## MATERIALS AND METHODS

**Patients**

Of the 43 Chinese NHL patients included in this study, 37 had B-cell neoplasm and 6 had T-cell neoplasm. All the patients were admitted to our hospital between August 2003 and December 2008. At the time of diagnosis the median age of the 30 male and 13 female patients was 51 years (range: 10-81 years). NHL was diagnosed according to the World Health Organization (WHO) classification system, based on morphological, immunophenotypical, and clinical features [3]. Initial clinical data for all patients were recorded, including age, sex, disease classification and stage, bone marrow infiltration, karyotype, serum p53 protein concentration, mortality, and follow-up. The study protocol was approved by the Ethics Committee and was performed in accordance with the Declaration of Helsinki. All the patients provided written informed consent to participate in the study. 

**Enzyme-linked immunosorbent assay and research into NHL**

Blood samples were collected from each patient. Peripheral blood and/or bone marrow aspirate smears stained with Wright-Giemsa were analyzed and classified according to the WHO classification for NHL [3]. Suspensions were collected from bone marrow aspirate material and cultured in RPMI 1640 medium supplemented with 20% fetal calf serum, penicillin-streptomycin, minimum essential medium vitamins, and glutamine at 37 °C for 24 h. Cells were exposed to colcemid overnight, followed by hypotonic treatment (KCL 0.075 M) for 30 min, and then fixation in ethanol and acetic acid (3:1). Chromosome analysis was based on reverse heat Giemsa (RHG)-banded metaphases. Chromosomal abnormalities were defined according to the 2005 International System for Human Cytogenetic Nomenclature [4]. The immunophenotype of each patient was confirmed via paraffin section immunoperoxidase, as previously described [5]. Serum samples were collected upon admission—prior to the administration of any treatment. Enzyme-linked immunosorbent assay (ELISA) for quantitative detection of human serum p53 protein was performed, according to the manufacturer’s instructions (Bender MedSystems, Germany). 

**Stage-modified international prognostic index (IPI)**

The stage-modified IPI was designed according to the IPI for which the original Ann Arbor stage II was substituted by the Lugano staging system for NHL. 

**Response criteria**

Response to treatment was evaluated based on imaging studies. Response criteria were defined according to International Working Group recommendations. CR was defined as the complete disappearance of all physical and radiological evidence of disease for ≥4 weeks. Biopsies of lymphomas were performed to confirm CR. Patients without CR at the end of treatment were considered treatment failures. OS was defined as the time from the date of diagnosis to the date of the final follow-up or time of death due to any cause.

## STATISTICAL ANALYSIS

Fisher’s exact test and logistic regression models were used to determine if any of the study parameters (age, sex, disease classification and stage, bone marrow infiltration, karyotype, and serum p53 protein) were independent prognostic factors of the overall response rate (CR + PR). Survival data were analyzed according to the Kaplan-Meier method, and were compared using the log-rank test. Multivariate regression analysis using the Cox proportional hazard model was used to identify risk factors on OS. Data were analyzed using SPSS v.12.0. A P value less than 0.05 was considered statistically significant.

## RESULTS

**Cytogenetic findings**

**Figure 1:**
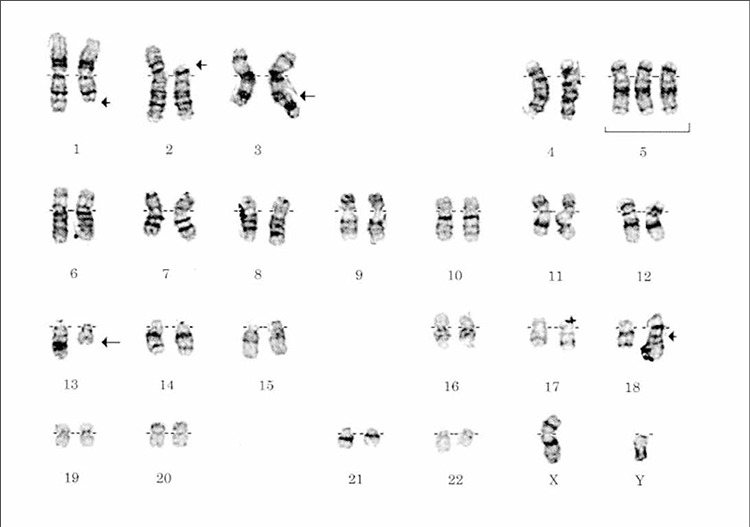
DLBCL karyotypes: 47; XY; del(1)(q41); l(2;18)(p13;q23); t(3;13)(q27;q14); +5[3]/46; XY[47].

Chromosomal abnormalities were observed in 19 patients and are summarized in Table 1. Abnormalities were taken into consideration if they were observed in a majority of abnormal cells or were known to be associated with a particular subtype of NHL. The main abnormalities showed a degree of association with the underlying diagnosis, although a few exceptions were apparent. The following translocations were observed: t(14;18)(q32;q2l) in 6 of 15 patients with diffuse large B-cell lymphoma (DLBCL); t(14; 18) in 3 of 7 patients with follicular lymphoma (FL); (q32;q21)t(11;14) in 2 of 3 patients with mantle cell lymphoma (MCL); t(11;18)(q21;q21) in 2 of 5 patients with Marginal zone b-cell lymphoma (MZBL); de1(13)(q14q31) in 1 of 6 patients with small lymphocytic lymphoma (SLL); t(8;14)(q24;q32) in the 1 patient with Burkitt’s lymphoma (BL); +3, +5, and +X in 2 of 3 patients with angioimmunoblastic lymphoma (AIL); t(2;5) (p23;q35) in 1 of 2 patients with Ki-l+ anaplastic large-cell lymphoma (Ki-1 ALCL); inv(14)(q11;q32) in the 1 patient with adult T-cell lymphoma. Figure 1 shows the DLBCL karyotypes. In this case, the histology of the lymph node predominantly showed the presence of small-cleaved cells with sparsely spaced large cells (14;18) was detectable cytogenetically. 

**p53 Expression and NHL**

In total, p53 protein expression <0.35 U/mL was observed in 1 of the 6 cases (16.7%) of T-cell NHL and in 14 of the 37 cases (37.8%) of B-cell NHL. According to REAL classification, p53 protein expression <0.35 U/mL was observed in 6 of the 15 DLBL patients, 3 of the 6 (50%) SLL patients, the 1 (100%) BL patient, 2 of the 6 (33.3%) peripheral T-cell lymphoma (PTCL) patients, 1 of the 3 (33.3%) MCL patients, 3 of the 7 (42.9%) FL patients, and 1 of the 3 AIL patients (Table 2). There wasn’t a correlation between p53 protein expression status, and age, sex, disease stage, T/B-cell type, or IPI score. 

**Analysis of short-term curative effect**

 Following conventional chemotherapy, 9 patients achieved CR and 14 patients achieved PR. The overall response rate (CR + PR) was 53.49%. In all, 20 patients had disease progression during treatment. Significantly more patients with early-stage (stage I-II) lymphoma, normal karyotype, and a low p53 protein level had CR/PR than did those with advanced-stage (stage III-IV) lymphoma, cytogenetic abnormalities, and a high serum p53 protein level. Bone marrow infiltration was also a predictor of poor response, as indicated by Fisher’s exact test, although there wasn’t a significant difference in the rate of disease remission between male and female patients, younger and older patients (aged <70 vs. ≥70 years), or B-cell lymphoma and T-cell lymphoma patients. Table 3 summarizes patient characteristics and Fisher P values associated with the overall response rate. 

Advanced-stage, bone marrow infiltration, and cytogenetic abnormalities were observed to be adverse prognostic factors based on univariate analysis, but not according to multivariate analysis; however, the serum p53 protein level was an independent prognostic factor for disease remission, based on the logistic regression model. The most likely cause of this is the existence of multicollinear relationships (high degree of correlation [correlation coefficient >0.7] between these independent variables) between the 4 variables mentioned above. As such, tumor stage, karyotype, and bone marrow infiltration might be considered prognostic factors that affect the overall response rate and the p53 protein level. The results of multivariate analysis using the logistic regression model are summarized in Table 4. 

**Analysis of long-term curative effect**

**Figure 2:**
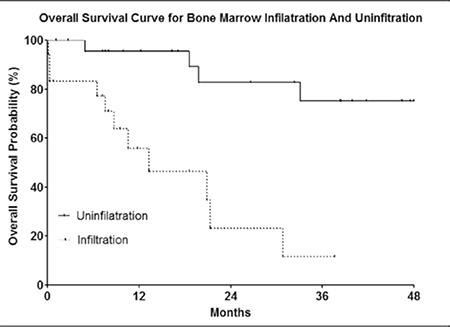
OS curve for bone marrow infiltration and noninfiltration.

**Figure 3:**
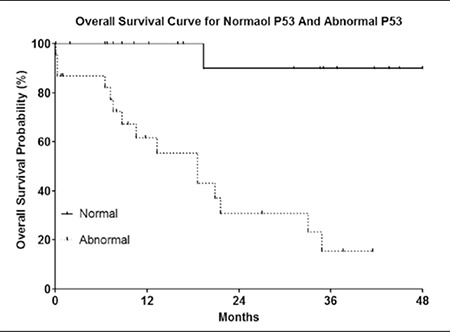
OS curve for low p53 protein level and high p53 protein level.

**Figure 4:**
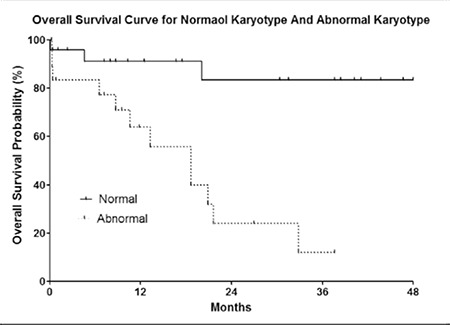
OS curve for normal and abnormal karyotypes.

OS was defined as the time from the date of diagnosis to the last follow-up evaluation or time of death due to any cause. Median survival time (MST) was 33 months. Bone marrow infiltration, cytogenetic abnormalities, and a low serum p53 protein level were all strongly associated with OS (Figures 2,3,4). The log-rank P values for OS are shown in Table 5. Log-rank testing showed that being female had a positive effect on prognosis (P = 0.0107). OS was significantly longer in patients with early-stage (stage I-II) lymphoma than in those with advanced-stage (stage III-IV) lymphoma; however, there wasn’t a significant difference in OS between younger (<70 years) and older (≥70 years) patients, or between patients with T-cell and B-cell lymphoma. 

Multivariate analysis using the Cox proportional hazard regression model was performed to assess the effect of the factors mentioned above on OS (Table 6). The data showed that sex and the p53 protein concentration affected OS, whereas advanced-stage lymphoma, bone marrow infiltration, and cytogenetic abnormalities were not significant prognostic factors. Multicollinearity may be the most important reason, and therefore the 3 variables were still significantly associated with tumor-related OS.

## DISCUSSION

A number of recurrent chromosomal abnormalities associated with histopathological subtypes and clinical outcomes have been identified in NHL [6]. Translocation t(14;18) and t(8;14) are strongly associated with FL and BL, t(11;14) is associated with MCL, and t(2;5) is associated with anaplastic large-cell lymphoma, and 3q27 abnormalities are associated with DLBCL [7]. Some chromosomal aberrations are associated with significantly poor prognosis; for example, rearrangement of 8q24, +7q, and del (13q) are independently associated with significantly shorter event-free survival in DLBCL, whereas del (13q) and +7q have a similar effect in DLBCL and BL [8]. In the present study 19 of the 43 NHL patients had cytogenetic abnormalities. It is difficult to assess the prognostic significance of individual cytogenetic alterations in a small patient cohort with a short follow-up period; however, the present findings strongly support the concept that chromosomal aberrations noted at the time of diagnosis are useful for predicting the overall response rate and/or OS using univariate and multivariable analysis. 

Chromosomal abnormalities were also observed in some patients with normal morphological characteristics, suggesting that bone marrow aspirate smears from a single puncture site may be of limited value in determining if the bone marrow is involved. Chromosomal analysis could be used as a supplementary technique. Another advantage of cytogenetic analysis is its ability to detect balanced translocations that would otherwise be missed by some DNAbased techniques [9]. Thus, it is necessary to perform cytogenetic examination to obtain useful information for diagnosis, staging, and prognostication. 

Molecular analysis of chromosome rearrangements has resulted in identification of several genes directly implicated in the biology of NHL; however, only with cytogenetic analysis will it become possible to identify additional events that could lead to further characterization of these genetic transpositions. Studies based on chromosome painting and in situ hybridization with breakpoint-specific probes may help elucidate the nature of these complex translocations and the genes involved in the secondary events. A sequential study may also establish the relative importance of these rearrangements to different stages of the lymphoma-leukemia development process and define the roles of the additional abnormalities described herein. 

The p53 gene is a cancer suppressor gene located at chromosome 17p13.1. It encodes a 53-kDa nuclear phosphoprotein (p53 protein). It acts as a negative regulator of the cell cycle [10]. It has been shown that p53 protein is activated in response to DNA damage and oncogenic stress via 2 distinct signaling pathways involving kinase-mediated phosphorylation of p53 protein by ATM/CHK2/1 cascade and inhibition of MDM2 via p19Arf, which results in p53 protein stabilization [11,12,13,14]. Solid tumors, including non-small-cell lung cancer, breast cancer, colorectal cancer, osteosarcoma, bladder cancer, and prostate cancer, exhibit increased apoptosis in response to Advexin [15]. Because serum p53 protein can be found in a small percentage of normal controls, its presence is not considered a diagnostic marker of cancer. Whether its serum concentration should be used as a screening marker or a predictive factor for the development of cancer remains to be determined. It has been reported to be variably present in the sera of patients with various malignancies. Several investigators have reported that the mean preoperative serum concentration of p53 protein in patients with head and neck squamous cell carcinoma was significantly higher than that in healthy controls. Higher serum p53 protein levels were also observed in lung cancer, pancreatic carcinoma, and colorectal cancer patients than in normal controls [16,17,18,19]. 

In the present study p53 protein was observed in the sera of NHL patients, which confirmed its value as a marker of the p53 concentration in NHL. The present data show that over-expression of p53 protein (≥0.35 U/ mL) was associated with a lower CR/PR rate and poor OS. Although the following conclusion cannot be considered definitive because of the small sample size, the findings suggest that serum p53 protein may be a potential prognostic marker for NHL. Unlike staging, sex is not a conventional means of assessing disease severity, and there is no consistent concept of its role in the prognosis of NHL. The present data show that female gender was positively correlated with longer OS than male gender, but this gender difference must be investigated further by studies with larger samples. 

**Conflict of Interest Statement **

None of the authors have any conflicts of interest, including specific financial interests, relationships, and/or affiliations, relevant to the subject matter or materials included.

## Figures and Tables

**Table 1:**
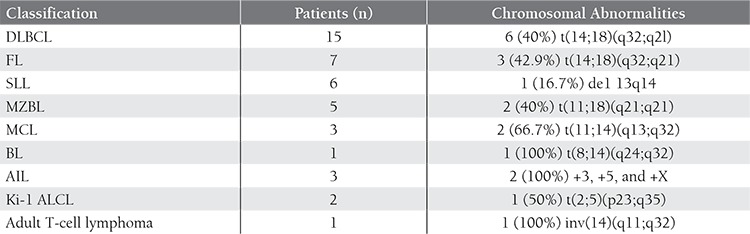
Cytogenetic data.

**Table 2:**
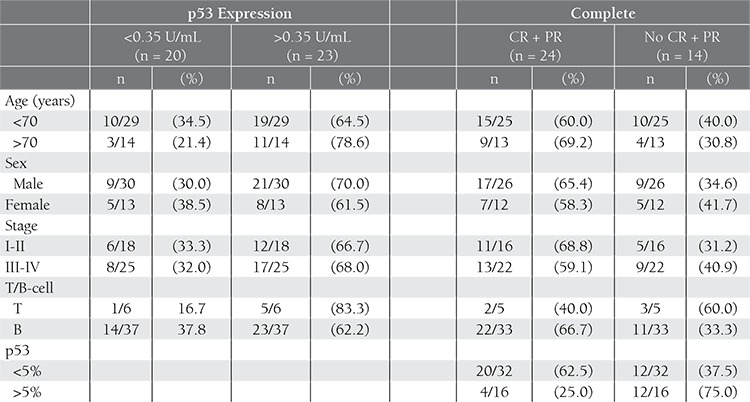
Characteristics in nodal NHL patients, according to p53 protein expression and response to chemotherapy.

**Table 3:**
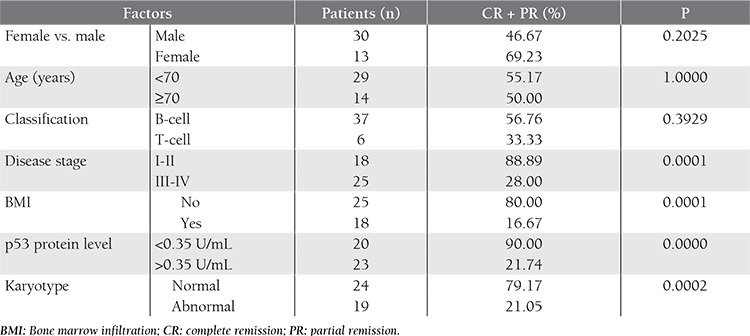
Fisher’s P values for risk factors associated with the overall response rate.

**Table 4:**
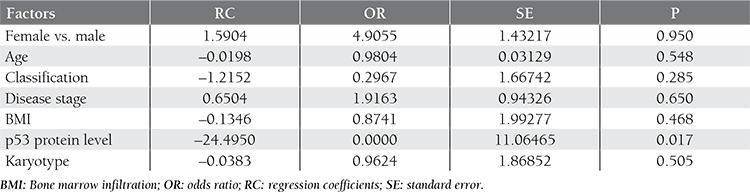
Results of multivariate analysis using a logistic regression model.

**Table 5:**
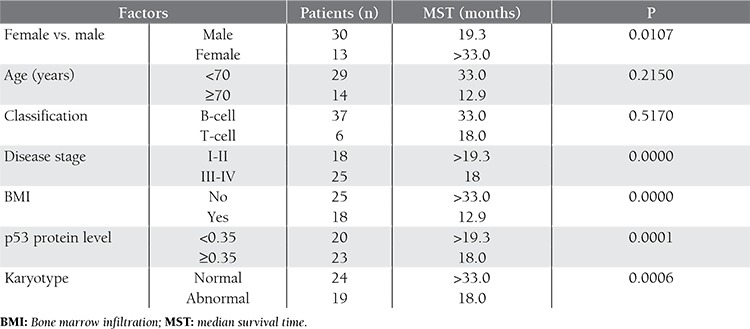
Prognostic factors and log-rank P values associated with OS.

**Table 6:**
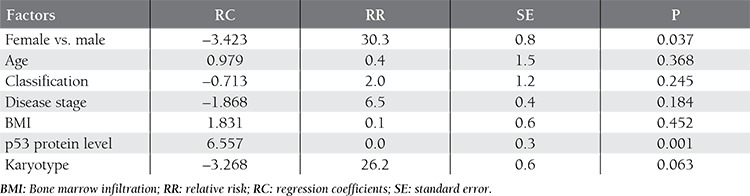
Multivariate analysis of OS based on Cox regression model means.
